# Co-production as an Emerging Methodology for Developing School-Based Health Interventions with Students Aged 11–16: Systematic Review of Intervention Types, Theories and Processes and Thematic Synthesis of Stakeholders’ Experiences

**DOI:** 10.1007/s11121-020-01182-8

**Published:** 2020-11-25

**Authors:** Hayley Reed, Danielle Couturiaux, Marianne Davis, Amy Edwards, Edward Janes, Hyun Sue Kim, G. J. Melendez-Torres, Simon Murphy, Torill Alise Rotevatn, Jesse Smith, Rhiannon Evans

**Affiliations:** 1 DECIPHer, UKCRC Centre of Excellence, Cardiff University, 1-3 Museum Place, Cardiff, CF10 3BD UK; 2 Public Health and Epidemiology Group, Department of Health Science and Technology, Aalborg University, Niels Jernes Vej 14, 9220 Aalborg East, Denmark

**Keywords:** Co-production, Intervention development, School health, Students, Systematic review, Thematic synthesis

## Abstract

Co-production affords an intervention’s target population the opportunity to participate in intervention theory decision-making during the development process. This addresses the over-reliance on developing interventions through academic theories which can be devoid of contextual understanding and result in challenges to implementing school-based health programmes. There is an emergent empirical literature on co-producing school-based health interventions, but an understanding of appropriate theoretical types and processes and stakeholders’ experiences is lacking. Through the conduct of a systematic review, this study seeks to understand the types and underlying theories and processes for co-production in school-based health interventions with students aged 11–16. A thematic synthesis explored stakeholders’ experiences of the different types of co-production. A systematic search of five electronic bibliographic databases, citation tracking of included studies, and consultation with an expert international panel were employed. Of 27,433 unique papers, 30 papers representing 22 studies were retained to describe types, and 23 papers of 18 studies used to synthesise stakeholders’ experiences. Three types were identified: external, individual-level, and system-level capacity-building. Whilst this review showed variability in co-production types, stakeholders involved and processes, shared functions were identified. Students’, school staff, facilitators’ and researchers’ experiences in terms of acceptability, feasibility and undertaking decision-making are discussed. Recommendations for conceptualising and reporting co-production and process evaluations of co-produced school-based health interventions are highlighted.

**Supplementary Information:**

The online version contains supplementary material available at 10.1007/s11121-020-01182-8.

Schools have been identified as key intervention settings to promote adolescent health (Bonell et al. 2014). However, evaluation of school-based interventions report mixed and often limited effectiveness, irrespective of whether they are monocomponent interventions focused solely on providing health education (Werner-Seidler et al. 2017) or utilise a multicomponent approach aligned to the Health Promoting Schools (HPS) framework (Langford et al. 2014). Integrated process evaluations indicate barriers to implementation that may compromise effectiveness, including lack of practical and philosophical fit with the context and limited responsiveness to individual school needs (Evans et al. 2015; Humphrey et al. 2015). It’s recognised interventions have been based too often on established academic theories which are devoid of contextual understanding (Moore and Evans 2017).

This has led to the reconsideration of intervention development processes to attend to context (Craig, 2018) through foregrounding stakeholder co-production (Moore and Evans 2017; Moore et al. 2019; Hawe et al. 2009). This is believed to lead to interventions based on more contextualised theories (Moore et al. 2019) which in turn increases the likelihood of intervention relevance, implementation and better outcomes (Craig et al. 2008). Recent intervention development examples (Hawkins et al. 2017), frameworks (Wight et al. 2016) and a taxonomy of health guidance (O’Cathain et al. 2019) give an overall direction on stakeholder co-production. Specifically in school settings, the range of interventions that encourage stakeholder co-production is growing, with the evidence base for effectiveness emergent but promising (i.e. Bonell et al. 2018; Ozer and Douglas 2013). However, co-production is a complex, egalitarian approach which involves a range of stakeholders and processes, often conflated to integrating different communities to work together. The crux, though, is to involve those traditionally excluded in key decision-making processes (Williams et al. 2020).

Students are one such group, and the primary population to involve in theory articulation for school-based interventions. The drive for involving children propagates from several sources. The political and legal status of children, drawn from the Convention on the Rights of the Child (United Nations 1989), outlines children’s right to be involved in decision-making about matters that affect them. The ‘new sociology of childhood’ recognises even very young children as competent ‘co-constructors of knowledge, identity and culture’ (Dahlberg et al. 2007) and experts in their own lives, with first-hand insights which adults are not privy to (Clark and Statham 2005). The HPS framework (WHO 1996) acknowledges students’ capability to be part of school decision-making, with democratic health education supporting this through the development of student action competence (Jensen 1997).

The key decision-making processes within intervention development, and this review, have been set as problem-setting and solving (Bond et al. 2001; Hawe et al. 2000). So stakeholders identify and understand the contextually situated drivers of problems and develop theory and activities to redress them (Moore and Evans 2017; Moore et al. 2019; Hawe et al. 2009).

Despite an increase in co-produced interventions and associated evaluation studies, there remains equivocality in regard to (i) the types, underlying theories and processes currently used to involve stakeholders in co-production within secondary schools, and (ii) stakeholders’ experiences of different types. This review addresses these gaps through systematically reviewing and synthesising extant research on co-produced school-based health interventions with students aged 11–16. Interventions must fall within the following:

1. Involvement is conducted within the school context. Secondary schools vary contextually so situating co-production within individual settings allows stakeholders to generate school-specific interventions responsive to student and staff needs.
2. Interventions are developed based on the views of those who will use them. At a minimum, this must be school students as the recipients of school-based health promotion interventions, omitting interventions where only adults contribute to co-production processes.
3. Stakeholders are involved iteratively in problem-setting and solving.

The review questions are:

1. What are the types of co-production currently utilised in developing school-based health interventions, and what are their underpinning theories of change and processes?
2. What are stakeholders’ experiences of these co-production types?

Effectiveness questions were not included as scoping showed few outcome evaluations.

## Methods

### Protocol and Registration

The systematic review protocol was registered with PROSPERO (CRD42018090920) and is reported in accordance with PRISMA (Shamseer et al. 2015).

### Eligibility Criteria

To be eligible for this review, papers needed to meet the following criteria: (i) population: young people aged 11–16, either as a subpopulation or as the whole population; (ii) intervention: fall within the review’s remit of intervention co-production, namely be conducted in schools, involve students, and include problem-setting and solving; (iii) setting: secondary school context (or international equivalent); (iv) outcome: violence and aggression, mental health and wellbeing, and/or substance use as a primary outcome, as review scoping indicated that these were the most productive areas of development around co-production; (v) data: a range of studies with co-produced interventions where qualitative data about the processes and stakeholders’ experiences were available in study documents; (vi) study: any country; (vii) language: published in English; (viii) date: published between 1986 and the date searches were conducted (February 2018) to coincide with the Ottawa Charter for Health Promotion which foreground stakeholder involvement (WHO 1986).

### Information Sources and Searches

Searches were conducted in five bibliographic databases most relevant to school-based health: Medline and PsycINFO (Ovid); Embase; ASSIA; and ERIC. The search was developed and refined in Embase (available online) before being adapted to the functionality of each database. Supplementary searching was conducted by consulting a panel of international experts, citation tracking of included studies and contacting study authors for further papers describing co-production processes and/or assessing stakeholders’ experiences.

### Study Selection

Retrievals were exported into Endnote for de-duplication before being uploaded to the Rayyan QCRI web application (Ouzzani et al. 2016). Study titles were screened by one reviewer to identify clearly irrelevant retrievals which were verified by a second reviewer. Two reviewers independently screened abstracts and then full papers. A third reviewer resolved conflicts.

### Data Extraction

A standardised extraction pro-forma was developed in Excel and piloted with a subset of included studies before being confirmed by the review team. Abstracted study characteristic items were as follows: author; publication; date; country; publication type; study aims; method; intervention characteristics; primary and secondary outcomes and process outcomes. Abstracted co-production items were the following: theoretical underpinning of co-production; stakeholders involved; recruitment; structure; development; problem-setting and solving processes undertaken and resultant health promotion activity adoption and implementation. Two reviewers independently abstracted data from a random sample of 10% of included papers, with the remainder extracted by one reviewer and checked for accuracy by a second.

### Quality Assessment

The EPPI Centre health promotion review criteria (e.g. Jamal et al. 2013; Rees et al. 2009) were used to assess the trustworthiness and relevance of study data. Trustworthiness assessed study sampling; data collection and synthesis; the extent findings are grounded in data. Relevance assessed the depth and breadth of co-production findings and whether accounts involved multiple co-production stakeholders. Two reviewers independently appraised studies, rating each item as ‘low’, ‘medium’ or ‘high’. Discrepancies were resolved through discussion. Quality assessment ratings varied (available online). Ratings were not employed as an inclusion criterion but as markers for the level of contribution papers had on syntheses.

### Syntheses

Findings were synthesised in two discrete, consecutive forms to address research questions.

#### Co-production Types and Underlying Theories and Processes

To differentiate types, the first author considered similarities and differences between studies through reading and re-reading the abstracted co-production data in Excel. Initial ideas about types were presented to two other team members who helped to clarify and refine types and their links to theory. To strengthen validity, two team members were briefed on co-production types and independently classified studies. To synthesise co-production processes, extracted data were formed into logic models for each co-production type by the first author (available online).

#### Stakeholder Experiences

 NVivo software was used to code stakeholder experiences. The first author coded all relevant sections of papers, noting some studies linked to multicomponent interventions where co-production was just one component. One-third of the papers were second-coded by another author to check for accuracy. Both coders used the following sequential steps. Data was coded into the different co-production types and then further coded into the different stakeholders present for that co-production type. Different stakeholders were not differentiated by the research team but as detailed in studies. Then, based on thematic synthesis (Thomas and Harden 2008), each line of papers linked to co-production was coded descriptively and memos were developed to summarise the themes found. Further memos were created to generate analytical themes. Drafts of results were presented to two team members who discussed and refined syntheses.

## Results

### Included Studies

**Fig. 1:**
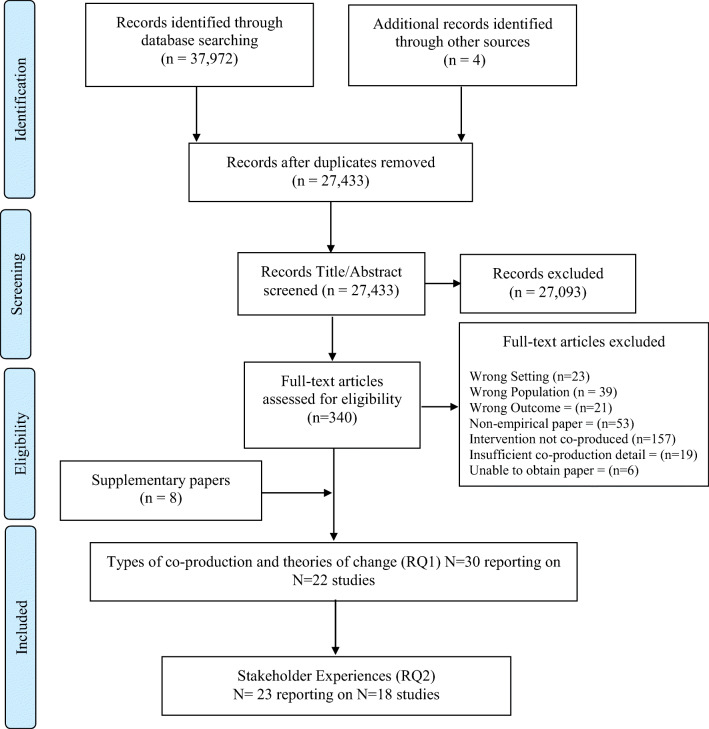
PRIMSA Flowchart of study selection

Study screening and retrieval are presented in Fig. 1. A total of 27,433 unique papers were retrieved including 4 expert recommendations. Following title and abstract screening, 340 papers progressed to full-text screening, with 22 papers meeting the inclusion criteria for the review. Eight supplementary papers were identified that provided further co-production data for seven included studies. In total, 30 papers reporting on 22 studies were included that addressed RQ1. A subset of these studies (23 papers reporting on 18 studies) addressed RQ2.

### Study Characteristics

An overview of the studies is given in Table 1. Twelve studies were conducted in the USA (Delara 2000; Miller 2010; Shriberg et al. 2017; Vaughn et al. 2013; Epstein 2007; Ozer et al. 2008, 2010, 2013; Soleimanpour et al. 2008; Mino 2003; Voight 2015; Youth In Focus 2002; Bell 2014; Bell et al. 2017), five in the UK (Paul et al. 2012, 2010; Bonell et al. 2015; Bonell et al. 2010a, b; Fletcher et al. 2015; Tew 2010), three in Canada (Hawe et al. 2015; Davison et al. 2011; Poulin and Nicholson 2005; Goodnough 2014), one in Australia (Bond et al. 2001; Glover et al. 2002) and one was pan-European (Simovska 2007; Simovska and Jensen 2008; Jensen et al. 2005). Nine of the 22 studies were case studies in single schools (Delara 2000; Paul et al. 2010, 2012; Vaughn et al. 2013; Voight 2015; Miller 2010; Goodnough 2014; Shriberg et al. 2017; Bell 2014; Bell et al. 2017); five were case examples of a single school selected to represent a multi-site study (Ozer et al. 2008, 2010) or studies where only one case example met the setting or outcome inclusion criteria (Tew 2010; Epstein 2007; Soleimanpour et al. 2008; Youth In Focus 2002); two were standalone process evaluations (Jensen et al. 2005; Simovska 2007; Simovska and Jensen 2008; Mino 2003); six were integrated process evaluations in pilot interventions with no control group (*n* = 2) (Bonell et al. 2010a, b; Davison et al. 2011; Hawe et al. 2015), a randomised pilot trial (*n* = 1) (Bonell et al. 2015; Fletcher et al. 2015), quasi-experimental designs (*n* = 2) (Poulin and Nicholson 2005; Ozer et al. 2013) or a cluster randomised control trial (*n* = 1) (Glover et al. 2002; Bond et al. 2001).

**Table 1:** Characteristics of studies included in the systematic review

Author and year	Country/ies	Methodology	Setting/s	Outcome	Component	Capacity-building type	Theory of change	Process frameworks
deLara 2000	USA	Case study	High school (*n* = 1)	School violence and safety	Mono	External	Action research, youth-based phenomenology and general system theory.	Action research
Paul et al. 2010	UK	Case study	Secondary school (*n* = 1)	Cyberbullying	Mono	External		Quality circles
Tew 2010	UK	Case example	Secondary school ^a^ (*n* = 1)	Mental health and wellbeing	Mono	External	Social and emotional aspects of learning and whole-school approaches.	PROGRESS programme
Paul et al. 2012	UK	Case study	Secondary school (*n* = 1)	Cyberbullying	Mono	External		Quality circles
Vaughn et al. 2013	USA	Case study	Elementary-middle school (*n* = 1)	Bullying and obesity ^b^	Mono	External	Community-based participatory research.	Concept mapping
Voight 2015	USA	Case study	Middle school (*n* = 1)	Bullying	Mono	External	Youth civic engagement and school climate	Freire dialogue circles
Jensen et al. 2005; Simovska 2007; Simovska and Jensen 2008	Czech Republic, Denmark, Sweden, Iceland, Portugal, Macedonia and Slovenia	Process evaluation	High school (*n* = 8)	I: youth, culture and alcohol II: Wellbeing and school environment	Mono	Individual-level	Health-Promoting Schools Framework and UN Convention on the Rights of the Child.	Investigation-vision- action-change
Epstein 2007	USA	Case example	High school (*n* = 1)	Student chosen gang violence & Teenage Pregnancy ^b^	Mono	Individual-level	Social action approach	Social action curriculum
Soleimanpour et al. 2008;	USA	Case example	High school (*n* = 1)	Student-chosen mental health ^b^	Mono	Individual-level	Community-based participatory research	Stepping stones
Youth In Focus 2002								
Ozer et al. 2008	USA	Case examples	Secondary school (n = 2)	Student-chosen e.g. gang and peer pressure	Mono	Individual-level	Youth-led participatory research	
Ozer et al. 2010	USA	Case example	Middle school (*n* = 1)	Student-chosen e.g. gang violence, and substance use	Mono	Individual-level	Youth participatory action research	
Ozer et al. 2013	USA	Process evaluation integrated into a quasi-experiment	High school (*n* = 4)	Student-chosen e.g. stress, school safety and cyberbullying	Mono	Individual-level	Community-based participatory research	
Miller 2010	USA	Case study	High school (*n* = 1)	Mental health and wellbeing	Mono	Individual-level	Participatory action research	Freirean Empowerment Education
Goodnough 2014	Canada	Case study	High school (*n* = 1)	student chosen smoking and school safety	Mono	Individual-level	Youth-adult community of practice	Youth action research
Shriberg et al. 2017	USA	Case study	Middle school (*n* = 1)	Bullying	Mono	Individual-level	Action research	Engage-identify-plan-act-reflect-sustain
Bond et al. 2001; Glover et al. 2002	Australia	Process Evaluation integrated into a cluster RCT	Secondary school (*n* = 12)	Mental health, bullying, and substance use	Multi	System-level	Attachment risk and protective factors, health-promoting schools framework and system capacity-building	Action research
Mino 2003	USA	Process evaluation	Middle school (*n* = 1)	Bullying, weapon carrying and violence	Mono	System-level		S.A.R.A. model
Poulin and Nicholson 2005	Canada	Process evaluation integrated into a quasi-experiment	Middle/senior school (*n* = 4)	Substance use and gambling	Mono	System-level	Cognitive dissonance, adolescent risk theory, social learning theory, stages of change model, co-operative participatory research and community capacity-building.	
Bonell et al. 2010a, b	UK	Process Evaluation integrated into intervention pilot	Secondary school (*n* = 2)	Substance use	Multi	System-level	Social psychology theory, pathways of school effects on drug use and health-promoting schools framework.	
Davison et al. 2011; Hawe et al. 2015	Canada	Process Evaluation integrated into an intervention pilot	High school (*n* = 1)	Mental health, bullying, substance use, and sexual health	Multi	System-level	Attachment and school connectedness, social development model, Health Promoting Schools Framework and social-ecological approach.	
Bell 2014; Bell et al. 2017	USA	Case study	Elementary-middle school (*n* = 1)	Wellbeing	Mono	System-level	Social and emotional learning implementation cycle, participatory culture-specific intervention model, public health problem-solving model for schools and school climate reform model.	SEL cycle
Fletcher et al. 2015; Bonell et al. 2015	UK	Process evaluation integrated into a pilot trial	Secondary school (*n* = 4)	Bullying, aggression and violence.	Multi	System-level	Theory of human functioning and school organisation and health-promoting schools framework	

^a^Only one case in the paper met the settings inclusion criteria. ^b^Only one case in the paper met the health outcome inclusion criteria

The interventions were either monocomponent (*n* = 18) (Delara 2000; Paul et al. 2010, 2012; Tew 2010; Vaughn et al. 2013; Voight 2015; Jensen et al. 2005; Simovska 2007; Simovska and Jensen 2008; Epstein 2007; Soleimanpour et al. 2008; Ozer et al. 2008, 2010, 2013; Miller 2010; Goodnough 2014; Shriberg et al. 2017; Mino 2003; Poulin and Nicholson 2005; Bell 2014; Bell et al. 2017; Youth In Focus 2002) meaning co-production was the only intervention element, or multicomponent (*n* = 4) (Bond et al. 2001; Glover et al. 2002; Bonell et al. 2010a, b; Davison et al. 2011; Hawe et al. 2015; Bonell et al. 2015; Fletcher et al. 2015), where a mixture of standardised and co-production components was used. There was an overlap in targeted health outcomes: 15 focused on aggression, violence and bullying (Delara 2000; Paul et al. 2010, 2012; Vaughn et al. 2013; Voight 2015; Epstein 2007; Ozer et al. 2008, 2010, 2013; Goodnough 2014; Shriberg et al. 2017; Bond et al. 2001; Glover et al. 2002; Mino 2003; Davison et al. 2011; Hawe et al. 2015; Bonell et al. 2015; Fletcher et al. 2015; Youth In Focus 2002); eight on mental health and wellbeing (Tew 2010; Jensen et al. 2005; Simovska 2007; Simovska and Jensen 2008; Soleimanpour et al. 2008; Miller 2010; Bond et al. 2001; Glover et al. 2002; Davison et al. 2011; Hawe et al. 2015; Bell 2014; Bell et al. 2017; Ozer et al. 2013) and seven on substance use (Jensen et al. 2005; Simovska 2007; Simovska and Jensen 2008; Ozer et al. 2010; Goodnough 2014; Bond et al. 2001; Glover et al. 2002; Poulin and Nicholson 2005; Bonell et al. 2010a, b; Davison et al. 2011; Hawe et al. 2015).

### Types of Co-production, Theories of Change and Processes

The review located three types of co-production for school-based health intervention with students aged 11–16 termed external, individual-level, and system-level capacity-building (see Table 1). Intervention inputs varied on the capacity-building undertaken so this was the criteria employed to distinguish types. Health promotion capacity is defined as the capability to ‘identify health issues and develop appropriate mechanisms to address them’ (Hawe et al. 2000), synonymous with the review’s definition of undertaking problem-setting and solving.

#### External Capacity-Building Co-production

This type involves generating co-production capacity outside of the school either through researchers increasing their knowledge of already established theories and process frameworks (Delara 2000; Paul et al. 2010, 2012; Vaughn et al. 2013; Voight 2015), or charity workers drawing on prior theory/research to develop a process framework (Tew 2010). These external stakeholders supported school stakeholders to undertake problem-setting and solving processes.

This type consisted of six interventions reported across six papers (Delara 2000; Paul et al. 2010, 2012; Vaughn et al. 2013; Voight 2015; Tew 2010) (Table 1). In studies that articulated a *theory of change* (*n* = 4), co-production was postulated to support stakeholders to voice their opinions on system change to improve health intervention success. Giving voice was articulated through involvement in research via action research (Delara 2000), community-based participatory research (Vaughn et al. 2013) and youth civic engagement (Voight 2015). Systems change was linked to school climate (Voight 2015), whole-school approaches (Tew 2010) and general systems thinking (Delara 2000). These theories briefly outlined the ethos of co-production; however, studies focused on process frameworks. The *process frameworks* (see Table 1) explicitly outlined the problem-setting and solving processes facilitators should run with stakeholders. Due to the heterogenous frameworks used, processes were very variable (see Figure 2).

Co-production took the *structure* of involving a core student group throughout (*n* = 3) (Paul et al. 2010, 2012; Voight 2015), or involving different stakeholders in different processes (*n* = 3), including students (Tew 2010; Vaughn et al. 2013; Delara 2000), staff members (Tew 2010; Vaughn et al. 2013; Delara 2000) and parents (Vaughn et al. 2013). Projects reporting on *recruitment* (*n* = 4) utilised staff nominations (Delara 2000; Paul et al. 2010, 2012; Voight 2015). One study used the *group development tasks* of ice breakers, agreeing ground rules and discussing participation and confidentiality (Paul et al. 2012).

*Problem-setting* was conducted through students collecting peer data (Paul et al. 2010), and/or deciding focal problems through consensus discussions (Voight 2015; Paul et al. 2010, 2012) in core student groups. Other studies used researcher conducted school surveys (Tew 2010; Vaughn et al. 2013), or small-scale surveys supplemented by student and staff interviews and focus groups (Delara 2000). *Problem-solving* processes were more varied and complex. In four studies, stakeholder groups discussed and decided on health activities (Paul et al. 2010, 2012; Voight 2015; Tew 2010). Three discussions were structured by Socratic questioning (Voight 2015), results of a school poll (Paul et al. 2010) and wellbeing pictures developed after analysing a school survey (Tew 2010). The other two were researcher-led, with one supporting stakeholders to brainstorm solutions for researchers to produce concept maps (Vaughn et al. 2013), whilst the other analysed survey, interview and focus group data to produce activity recommendations which were student verified (Delara 2000).

Only three papers referenced *adoption* of decided health activities with all noting that school administration made the final adoption decisions, supported by a report (Paul et al. 2012), student presentations (Voight 2015), or student/staff developed problem pictures (Tew 2010). Activities were briefly noted as a mixture between student-led classroom initiatives, such as developing board games, and school-level, i.e. a student leadership scheme (Paul et al. 2012). Another study noted multiple systemic activities, including student lesson observations and staff relationship training, and modifying school timings and behaviour systems (Tew 2010). The last study reported numerous recommended changes such as community fundraisers but systemic changes, like making curricula more experiential, were not adopted (Voight 2015). No study detailed how, and if, *implementation* was achieved. Outcome *evaluations* (*n* = 2) collected pre and post socio-emotional competency for co-production students, and a student survey of school climate and pro and antisocial behaviour (Voight 2015); the second study used a reduction in behaviour incidents (Paul et al. 2010).

#### Individual-Level Capacity-Building Co-production

This type involved the delivery of training/curricula to students, sometimes via school teachers, before (Miller 2010; Goodnough 2014; Ozer et al. 2008, 2010, 2013; Jensen et al. 2005; Simovska 2007; Simovska and Jensen 2008; Epstein 2007) or during (Soleimanpour et al. 2008; Youth In Focus 2002; Shriberg et al. 2017) facilitated process cycles. Researchers (Jensen et al. 2005; Simovska 2007; Simovska and Jensen 2008; Miller 2010; Goodnough 2014; Shriberg et al. 2017) or youth workers (Epstein 2007; Soleimanpour et al. 2008; Ozer et al. 2008, 2010, 2013; Youth In Focus 2002) developed curricula, with five combinations of delivery to recipients (see Figure 3). This upskilled students to undertake problem-setting and solving processes to decide on health activities, including students deciding the form of project processes i.e. what problem-setting methods to use.

This type consisted of nine interventions reported across 12 papers (Jensen et al. 2005; Simovska 2007; Simovska and Jensen 2008; Epstein 2007; Soleimanpour et al. 2008; Ozer et al. 2008, 2010, 2013; Miller 2010; Goodnough 2014; Shriberg et al. 2017; Youth In Focus 2002) (Table 1). The *theories of change* articulated how developing students to become school researchers or change leaders could allow them to address school health issues and change their own health behaviours. Interventions were influenced by theories which advocated for student involvement in the following: (i) research (*n* = 7), termed community-based participatory action research (Soleimanpour et al. 2008; Ozer et al. 2008, 2010, 2013; Youth In Focus 2002), participatory action research (Miller 2010; Shriberg et al. 2017) and youth-led action research (Goodnough 2014); (ii) addressing social injustice through the social action approach (Epstein 2007); (iii) promoting health, through the HPS framework (Simovska 2007; Jensen et al. 2005; Simovska and Jensen 2008).

Therefore, curricula focused on teaching students research skills (*n* = 6) (Goodnough 2014; Soleimanpour et al. 2008; Ozer et al. 2008, 2010, 2013; Youth In Focus 2002; Miller 2010); leadership skills to take collective action (*n* = 2) (Epstein 2007; Shriberg et al. 2017); organisational change (*n* = 5) (Soleimanpour et al. 2008; Youth In Focus 2002; Shriberg et al. 2017; Ozer et al. 2008, 2010, 2013); or raising teacher awareness of the participatory framework to enable them to support students (*n* = 1) (Simovska and Jensen 2008; Simovska 2007; Jensen et al. 2005). Six projects acknowledged student learning was situated through social participation in research (Jensen et al. 2005; Simovska 2007; Simovska and Jensen 2008; Epstein 2007; Ozer et al. 2008, 2010, 2013; Goodnough 2014) which was a paramount aim to collecting rigorous data for problem-setting and solving decision-making.

All studies named *process frameworks* (see Table 1) with stages that structured co-production processes. The *structure* of co-production was either small student groups (Shriberg et al. 2017; Goodnough 2014; Soleimanpour et al. 2008) or classes (Simovska 2007; Jensen et al. 2005; Simovska and Jensen 2008; Epstein 2007; Ozer et al. 2008, 2010, 2013; Miller 2010). Group-level *recruitment* was via staff nominations (Shriberg et al. 2017), student applications (Soleimanpour et al. 2008) or the school council (Goodnough 2014). At the class-level, students took electives (Ozer et al. 2008, 2010, 2013; Epstein 2007) or teachers selected classes into projects. Studies (*n* = 7) outlined the *group development tasks* of ice breakers (*n* = 3) (Miller 2010; Youth In Focus 2002; Soleimanpour et al. 2008; Shriberg et al. 2017), communication skills/active listening (*n* = 4) (Ozer et al. 2008, 2010, 2013; Epstein 2007), goal setting (*n* = 2) (Shriberg et al. 2017; Epstein 2007) or developing ground rules (*n* = 1) (Shriberg et al. 2017).

As these studies were linked theoretically and procedurally through the lens of action research, students often decided project processes. Hence, *problem-setting* processes allowed students free reign to choose problem targets (Ozer et al. 2008, 2010, 2013; Epstein 2007; Goodnough 2014; Soleimanpour et al. 2008), or gave broad health theme focuses (Simovska 2007; Jensen et al. 2005; Simovska and Jensen 2008; Miller 2010), with only one constraining the focus to bullying (Shriberg et al. 2017). Two studies led students to consider their own ideas in prescribed group discussions (Epstein 2007) and photography (Miller 2010) to understand the school health problems, whereas seven projects allowed students to decide how to problem set and who to involve (Jensen et al. 2005; Simovska 2007; Simovska and Jensen 2008; Soleimanpour et al. 2008; Ozer et al. 2008, 2010, 2013; Goodnough 2014; Shriberg et al. 2017). Students choose from a myriad of data collection forms, including surveys, interviews, PhotoVoice and mapping, with school peers (Soleimanpour et al. 2008; Ozer et al. 2008, 2010, 2013; Goodnough 2014; Jensen et al. 2005; Simovska 2007; Simovska and Jensen 2008), staff (Goodnough 2014; Shriberg et al. 2017) or students in other project schools (Simovska 2007; Jensen et al. 2005; Simovska and Jensen 2008).

Descriptions of *problem-solving* processes were brief. All studies noted using student group discussions, with a few (*n* = 4) structuring discussions through the prior problem-setting research (Simovska and Jensen 2008; Simovska 2007; Jensen et al. 2005; Goodnough 2014), facilitator questioning (Miller 2010), or facilitator scaffolding (Ozer et al. 2013). *Adoption* was frequently considered by decision-makers once students had presented their recommendations (Shriberg et al. 2017; Goodnough 2014; Ozer et al. 2008, 2010; Soleimanpour et al. 2008; Miller 2010), although one study allowed schools to individually decide *adoption* processes (Jensen et al. 2005; Simovska 2007; Simovska and Jensen 2008). Two studies led to direct adoption without a formalised process, but facilitators constrained actions to feasible, student-led activities like mural painting (Epstein 2007; Ozer et al. 2013). Three studies did not detail *implementation* of student ideas (Goodnough 2014; Shriberg et al. 2017; Miller 2010), whereas others noted multiple changes to schools and playgrounds, tackling recycling and litter, developing a school council and writing to the Mayor to affect community change (Simovska 2007; Jensen et al. 2005; Simovska and Jensen 2008; Ozer et al. 2008, 2010, 2013; Soleimanpour et al. 2008). No study discussed assessing *implementation*. *Evaluation* in one study measured the differences in socio-political skills, motivation to influence, participatory behaviour and perceived control between intervention and control students (Ozer et al. 2013).

#### System-Level Capacity-Building Co-production

This type involved the development of research action groups (RAGs) consisting of multiple school and external stakeholders. The predominant focus was to ensure rigorous data collection about student health and school functioning, so RAGs could make informed problem-setting and solving decisions. RAGs were supported by three other inputs to aid processes (see Figure 4). External facilitators (*n* = 6) were either researchers (Bond et al. 2001; Glover et al. 2002; Bell 2014; Bell et al. 2017; Poulin and Nicholson 2005) or employees with previous teaching/facilitation experience (Davison et al. 2011; Hawe et al. 2015; Bonell et al. 2010a, b; Bonell et al. 2015; Fletcher et al. 2015), school-level data (*n* = 9) and/or intervention manuals (*n* = 3) (Bonell et al. 2015; Bonell et al. 2010a, b; Fletcher et al. 2015; Bond et al. 2001; Glover et al. 2002).

This type consisted of seven interventions reported across 12 papers (Bonell et al. 2015; Bonell et al. 2010a, b; Fletcher et al. 2015; Bond et al. 2001; Glover et al. 2002; Bell 2014; Bell et al. 2017; Davison et al. 2011; Hawe et al. 2015; Mino 2003; Poulin and Nicholson 2005) (Table 1). Generally, interventions utilised a pluralistic approach to layering formalised theory, conceptual models, process frameworks and/or prior research to articulate *theories of change*. For example, all multicomponent interventions (Bonell et al. 2015; Bonell et al. 2010a, b; Fletcher et al. 2015; Bond et al. 2001; Glover et al. 2002; Davison et al. 2011; Hawe et al. 2015) drew on previous interventions and were influenced by the HPS framework necessitating change at the individual, organisational and community levels. Co-production was positioned at the organisational level. Projects integrated individual and social psychology theories (Hawe et al. 2015; Bond et al. 2001; Poulin and Nicholson 2005; Bonell et al. 2010b) acknowledging co-production linked individual and organisation through i.e. school connectedness (Hawe et al. 2015; Bond et al. 2001). Sociological theories postulated decision-making groups change the health behaviours of those involved, and the resulting health activities affect population health (Bonell et al. 2015; Fletcher et al. 2015; Bonell et al. 2010b).

As only three studies utilised *co-production frameworks* (Bell 2014; Bell et al. 2017; Mino 2003; Bond et al. 2001; Glover et al. 2002)**,** problem-setting and solving processes often needed extricating. The only* structure* was development of RAGs. Interventions necessitated *recruitment* of school and external stakeholders like students, parents, governors, teaching staff, senior management and police, but did not always give recruitee data. One RAG involved only one student (Bell 2014; Bell et al. 2017), another advising six students minimum (Fletcher et al. 2015; Bonell et al. 2015; Davison et al. 2011; Hawe et al. 2015); again, some studies omitted student numbers (Bond et al. 2001; Glover et al. 2002). Recruitment processes were decided by schools (Poulin and Nicholson 2005; Davison et al. 2011; Hawe et al. 2015; Bonell et al. 2010a, b), through election or self-nomination (Bond et al. 2001; Glover et al. 2002; Mino 2003), principal nomination (Bell 2014; Bell et al. 2017) or a mixture of staff and student nomination (Bonell et al. 2015). *Group development tasks* of agreeing group rules and goals and electing roles were detailed in two studies (Bell 2014; Bell et al. 2017; Bond et al. 2001; Glover et al. 2002).

The health targets were pre-determined by research teams or funders. Hence, *problem-setting* involved developing an understanding of school context and how the target problem functioned there, aligning with a systems approach. Six projects prescribed utilising researcher needs assessments with student populations or 1-year group (Bonell et al. 2015; Bonell et al. 2010a, b; Fletcher et al. 2015; Bond et al. 2001; Glover et al. 2002; Bell 2014; Bell et al. 2017; Davison et al. 2011; Hawe et al. 2015; Mino 2003) and staff (Bell 2014; Bell et al. 2017). In addition, four projects undertook audits of current policies, programmes and practices (Bonell et al. 2015; Bonell et al. 2010a, b; Fletcher et al. 2015; Bond et al. 2001; Glover et al. 2002; Bell 2014; Bell et al. 2017; Davison et al. 2011; Hawe et al. 2015), one included mapping safety hotspots (Mino 2003), another PhotoVoice with students and staff social network analyses (Davison et al. 2011; Hawe et al. 2015), whereas one study relied only on RAG discussions (Poulin and Nicholson 2005).

Prioritisation involved ranking through the needs assessment survey (Bonell et al. 2010a, b), ranking exercises (Bond et al. 2001; Glover et al. 2002; Davison et al. 2011; Hawe et al. 2015; Bell 2014; Bell et al. 2017) or group discussion (Poulin and Nicholson 2005; Bonell et al. 2010a, b; Bonell et al. 2015; Fletcher et al. 2015; Mino 2003). Supplementary data was used (*n* = 2) in the form of school routine data (Bonell et al. 2015; Fletcher et al. 2015) and focus groups (Bell 2014; Bell et al. 2017) before prioritisation. *Problem-solving*, informed by problem-setting data, was achieved through RAG discussions with reference to checking theory, practice and research in two studies (Bell 2014; Bell et al. 2017; Bond et al. 2001; Glover et al. 2002).

*Adoption* and *implementation* of health activities were seamless in most studies as RAGs and facilitators remained to support this (*n* = 6) (Bonell et al. 2015; Bonell et al. 2010a, b; Fletcher et al. 2015; Bond et al. 2001; Glover et al. 2002; Davison et al. 2011; Hawe et al. 2015; Mino 2003; Poulin and Nicholson 2005), demonstrating delivering actions was intrinsic to this type. Activity funding was granted a priori in two studies (Bonell et al. 2015; Fletcher et al. 2015; Mino 2003), a priori and responsively (Bonell et al. 2010a, b), and post project (Bell 2014; Bell et al. 2017). Actions delivered were numerous ranging through socio-ecological levels i.e. delivering bullying workshops, student-led dramas, amending timetables, and school behaviour and pastoral policies, and holding a community conference; however, activity *implementation* was not assessed. Interventions *evaluated* school-level outcomes of the substantive project foci (*n* = 4) (Bond et al. 2001; Glover et al. 2002; Davison et al. 2011; Hawe et al. 2015; Bonell et al. 2015; Fletcher et al. 2015; Poulin and Nicholson 2005); intermediate outcomes on postulated co-production pathways (*n* = 2) (Bonell et al. 2010a, b; Bonell et al. 2015; Fletcher et al. 2015); RAG co-production levels of acceptability (Bonell et al. 2015; Fletcher et al. 2015); and social validity and acceptability (Bell 2014; Bell et al. 2017).

### Stakeholders’ Experiences

Co-production experiences were found in 23 papers reporting on 18 studies. Stakeholders represented were intervention developers (students, school staff, external partners like parents and facilitators) and those studying intervention development (researchers). Note co-production facilitators and researchers were sometimes the same individual. Views are separated by stakeholder where this was done in data sources. The analytical themes found were acceptability (how stakeholders received co-production); feasibility (stakeholders’ thoughts on how co-production interacted with context); and decision-making (stakeholders’ thoughts on developing and delivering co-produced health activities).

#### External Capacity-Building Co-production

Students’, school staff and facilitators’/researchers’ experiences were reported in three papers on three interventions (Paul et al. 2010, 2012; Voight 2015). Data was limited and different stakeholders’ views were not always separated.

Papers indicated only general statements about *acceptability*, such as student and staff feedback was positive (Paul et al. 2010, 2012), students felt empowered and projects achieved their aims (Paul et al. 2012). Acceptability was linked by all stakeholders to perceived benefits, such as improvements in student’s prosocial behaviours and better student-student and student-teacher relationships (Voight 2015). Student groups were mostly considered *feasible* within schools by staff and researchers. Two exceptions were that groups struggled to form cohesively, so facilitators needed to mediate between students (Paul et al. 2010), and projects were time-restricted when not scheduled into lessons.

Staff and researchers limited health activity *decision-making* to manageable (Paul et al. 2010), classroom-based changes (Paul et al. 2012), as they worried students would develop irresponsible activities outside of the scope of a school (Voight 2015). Researchers thought student ideas showed a propensity to understand the root causes in terms of individual behaviours rather than organisational influences, leading to implementing student-led activities not systemic change (Voight 2015). This may be ameliorated by having a range of stakeholders involved (e.g. Tew 2010), but no data was collected on this.

#### Individual-Level Capacity-Building Co-production

Students’, facilitators’ (teachers’ and youth workers’) and researchers’ experiences were reported in 11 papers of nine interventions (Jensen et al. 2005; Simovska 2007; Simovska and Jensen 2008; Epstein 2007; Soleimanpour et al. 2008; Ozer et al. 2008, 2010, 2013; Miller 2010; Goodnough 2014; Shriberg et al. 2017). Papers tended to privilege teacher, youth worker and researcher perspectives over students.

Students’ experienced this type as *acceptable* because they felt empowered, had project ownership and were afforded an opportunity to take part in decision-making during learning (Simovska 2007), research projects (Goodnough 2014; Shriberg et al. 2017; Ozer et al. 2013) and school change (Goodnough 2014; Epstein 2007; Ozer et al. 2013). They perceived more control over goal-setting (Ozer et al. 2013) than teachers, who were advisors (Ozer et al. 2013; Jensen et al. 2005) and trusted learning partners (Goodnough 2014). Taking photos (Miller 2010), selecting topics, developing questions and conducting quality interviews and surveys (Jensen et al. 2005; Soleimanpour et al. 2008; Shriberg et al. 2017; Ozer et al. 2008) were considered demanding tasks. Nevertheless, they felt they benefitted from increased health (Shriberg et al. 2017; Miller 2010; Simovska 2007; Jensen et al. 2005; Goodnough 2014) and research knowledge (Goodnough 2014), confidence (Jensen et al. 2005) and skills in leadership (Shriberg et al. 2017; Goodnough 2014), communication (Ozer et al. 2013), teamwork (Epstein 2007; Jensen et al. 2005) and problem-solving (Jensen et al. 2005).

Students raised *feasibility* concerns about discussing sensitive issues and having limited time. Discussing sensitive issues, i.e. gang affiliation, was problematic due to confidentiality and because students thought facilitators would judge them (Ozer et al. 2013). Time was important to develop the trusting student-facilitator relationships needed (Shriberg et al. 2017; Goodnough 2014), conduct research outside lesson time (Soleimanpour et al. 2008) and complete projects within a year which was not always achieved. Students accepted actions were long term though (Goodnough 2014) and were happy to forego *decision-making* by implementing previous cohort projects, if needed (Ozer et al. 2013).

Facilitators thought the democratic, transformative pedagogy underlying co-production was more *acceptable* than traditional, didactic health risk-based curricula (Simovska and Jensen 2008; Jensen et al. 2005; Epstein 2007). They recounted that student engagement varied by confidence (Jensen et al. 2005), motivation (Jensen et al. 2005; Epstein 2007) and skill acquisition (Simovska and Jensen 2008). This was affected by whether students had elected or understood project requirements before electing classes (Ozer et al. 2010; Jensen et al. 2005). Therefore, sustaining engagement in lengthy projects was necessary through encouragement (Simovska and Jensen 2008) and incentives (Soleimanpour et al. 2008). Teachers also reinforced student concerns about mastering skills (Jensen et al. 2005; Goodnough 2014), particularly due to short skill development times (Goodnough 2014); but acknowledged engagement leads to student personal growth (Epstein 2007; Simovska 2007), development of leadership (Shriberg et al. 2017; Goodnough 2014) and action competence (Simovska and Jensen 2008) and a wider awareness of health (Simovska and Jensen 2008).

Teachers expressed *feasibility* concerns as the new pedagogy involved high levels of commitment (Jensen et al. 2005), and they worried they were occasionally leading rather than scaffolding learning (Simovska 2007; Ozer et al. 2013). Some students needed additional support which was difficult in classrooms (Jensen et al. 2005). Initial training was important (Jensen et al. 2005); although ongoing external, scheduled and responsive support for research naïve facilitators was imperative to translate training into teaching (Ozer et al. 2008; Jensen et al. 2005) demonstrated when researchers observing classes were drawn in as co-teachers (Ozer et al. 2008). Ongoing support was especially necessary when only one teacher was involved as no peer support was available (Jensen et al. 2005). Overall, facilitators thought delivering curricula was more feasible in schools where the national curriculum aligned to problem-solving (Jensen et al. 2005); previous relationships existed between youth organisations and schools (Ozer et al. 2010); an established youth training workforce existed (Ozer et al. 2010); and lesson time was allocated (Simovska and Jensen 2008; Goodnough 2014; Soleimanpour et al. 2008; Jensen et al. 2005).

Facilitators noted challenges with implementing student ideas after *decision-making* (Simovska and Jensen 2008), especially in larger schools with fragmented teacher networks (Ozer et al. 2010) or where headteacher support was absent (Jensen et al. 2005). They thought lack of implementation led to future student disengagement so constrained issue selection and actions to realistic, short-term change (Ozer et al. 2010), attenuating this through ensuring students made micro-decisions i.e. data collection methods (Ozer et al. 2013).

Researchers thought the projects were *feasible* because students developed as cohesive groups (Epstein 2007), and built solid relationships with outside facilitators (Goodnough 2014). They only noted varying student interest as a function of social maturity, causing classroom disruption and sometimes the need to adapt co-production by recruiting smaller groups to continue class work (Ozer et al. 2010; Epstein 2007; Ozer et al. 2013). They also thought curricula were well delivered, but more difficult within schools without a tradition of empowerment (Shriberg et al. 2017), or those focused on improving educational standards (Ozer et al. 2008, 2010). They acknowledged school management involvement was needed due to lengthy delivery times (Ozer et al. 2010). To ensure project traction and school embeddedness, there was a demand for regular communication with administration (Shriberg et al. 2017; Ozer et al. 2008), linking to established school structures (Shriberg et al. 2017), and planning for students to take over unfinished projects (Ozer et al. 2008). Continuity was an issue due to teacher and student turnover (Shriberg et al. 2017), and youth organisation withdrawal after funding ceased (Ozer et al. 2008).

Researchers noted a number of issues with *decision-making*. Students choose activities that contested school-level policies (Ozer et al. 2008) or political and administrative functioning (Ozer et al. 2013); were too resource-intense (Ozer et al. 2008); or required change outside the school (Jensen et al. 2005). Therefore, ideas were not always adopted (Ozer et al. 2008; Ozer et al. 2013) and/or facilitators supported students to think of realistic, student-led actions deliverable within project time frames (Ozer et al. 2013; Soleimanpour et al. 2008), or ‘quick wins’ to maintain engagement and ensure implementation (Ozer et al. 2008; Goodnough 2014). Even when ideas were adopted, researchers questioned whether this was due to student recommendations, or a coincidental fit with educational system change (Ozer et al. 2010). A lack of idea implementation was considered a learning opportunity about democracy (Jensen et al. 2005), or curricula were adapted to incorporate lessons learnt (Ozer et al. 2008). Researchers concluded some projects emphasised developing responsible citizens, in lieu of actual school change (Epstein 2007). Students were not always aware of this, as they were not supported to critically evaluate project impact (Ozer et al. 2013).

#### System-Level Capacity-Building Co-production

Students’, school staff, facilitators’ and researchers’ experiences were reported in nine papers reporting on six studies (Bonell et al. 2015; Bonell et al. 2010a, b; Fletcher et al. 2015; Bond et al. 2001; Bell 2014; Bell et al. 2017; Davison et al. 2011; Mino 2003). Papers tended to be very comprehensive process evaluations, so provided extensive data from all stakeholders.

Students thought co-production was *acceptable* as it was a new and enjoyable experience (Mino 2003; Bonell et al. 2015), and because using a range of recruitment techniques meant RAGs were diverse (Bonell et al. 2015). RAGs and need assessments were perceived as key to hear all student voices (Fletcher et al. 2015), sometimes for the first time (Mino 2003). The lack of prior decision-making opportunities drove student participation (Fletcher et al. 2015; Bonell et al. 2015). One study highlighted RAGs were more acceptable than student councils as they were more representative and diverse, allowed younger students responsible roles, focused on true collaboration, real student issues, and resulted in an actual change (Fletcher et al. 2015; Bonell et al. 2015). Students felt a sense of being listened to, having improved self-regard and confidence, empowerment and school ownership, resulting in greater engagement in learning (Bonell et al. 2010a, b; Fletcher et al. 2015).

Students thought adult style meetings were *feasible***,** as even those initially reluctant to speak felt able to contribute over time (Bonell et al. 2010a). Students were split on holding meetings at lunch or after school though (Bonell et al. 2015). Taking part in *decision-making* allowed students to understand the complexities of school change, and teacher difficulties to implement change, leading to better student-teacher relationships (Bonell et al. 2010b).

Adult RAG members agreed about *acceptability*, giving examples of students who benefitted from i.e. improved self-regard (Bonell et al. 2010a, b). Students and staff also emphasised participation in resulting health activities had an additive beneficial effect (Bonell et al. 2010a, b; Bonell et al. 2015; Fletcher et al. 2015). They said everyone was listened to and could have their say; their inputs were valued and respected which demonstrated empowerment (Bell 2014; Bonell et al. 2010b; Bell et al. 2017). Overall, they thought acceptability was the highest when projects were congruent with needs (Fletcher et al. 2015; Bonell et al. 2015; Mino 2003), prior commitments (Mino 2003) or a desire for bottom-up change (Bell 2014; Bell et al. 2017). A focus on health was welcomed (Bell 2014; Bond et al. 2001) but community issues needed tackling too (Mino 2003; Bell 2014).

Adult RAG members discussed how external facilitators made projects more *feasible*. They guided processes (Bond et al. 2001), maintained project momentum (Bell 2014; Bond et al. 2001), acted as student advocates (Bonell et al. 2015) and provided an outside perspective (Bonell et al. 2010b; Bond et al. 2001) and link to support (Bond et al. 2001). Teachers thought they improved their capacity to truly collaborative with students (Fletcher et al. 2015; Bonell et al. 2015; Bell 2014; Bell et al. 2017), but a minority felt overwhelmed due to other work and personal pressures, or inexperience in processes like data analysis (Bell 2014). Additionally, needs assessments were considered imperative to *decision-making*, as they gave a bottom-up understanding of student issues (Bell 2014). Data supported RAGs to ensure schools did not dismiss important issues (Fletcher et al. 2015), including understanding and legitimatising known problems (Bell 2014; Bell et al. 2017; Fletcher et al. 2015; Bond et al. 2001), or discovering new ones (Fletcher et al. 2015; Bond et al. 2001; Mino 2003).

Facilitators thought *acceptability* would be attenuated as Inspectorates and parents may view data negatively (Bonell et al. 2015); however, senior staff opposed this, perceiving the data and the inclusion in interventions as evidence of school strengths (Bonell et al. 2010a; Fletcher et al. 2015). Senior staff thought projects appealed as they provided a contextually tailored intervention, resources such as finances and facilitator time, and fit with educational policies for student involvement (Fletcher et al. 2015) and health (Bonell et al. 2015; Bell 2014). They perceived intervention flexibility as advantageous as groups considered schools starting systems so they could build on prior work (Bond et al. 2001; Bonell et al. 2015) and the school ethos (Bonell et al. 2010a; Bonell et al. 2015), or try new activities (Bond et al. 2001; Bonell et al. 2015). One study noted management attributing benefits of increased attendance and positive Inspectorate feedback to the project (Bonell et al. 2015).

Adult RAG members and facilitators outlined conditions that did/could increase *feasibility*. School recruitment was necessary in the prior year as co-production and implementation were lengthy (Bonell et al. 2015); manuals were unwieldy, so facilitators were selective in their use (Bonell et al. 2010b); teaching/facilitation experience was important for facilitators (Fletcher et al. 2015; Bonell et al. 2015); and senior staff involvement was necessary for RAGs to progress (Bonell et al. 2010a; Mino 2003; Fletcher et al. 2015; Bond et al. 2001). A lack of implementation of health activities after *decision-making* was attributed to a disparity with schools’ ethos (Bonell et al. 2010a). It was thought implementation could be improved by integrating projects into School Improvement Cycles (Bonell et al. 2015).

Researchers agreed with the preceding experiences and summarised co-production themes. Projects were *acceptable* in schools of varying deprivation, inspection ratings and baseline contexts (Bonell et al. 2015; Fletcher et al. 2015; Bond et al. 2001; Bonell et al. 2010a, b). *Feasibility* was increased when school leaders committed to progressing projects (Bell 2014; Bond et al. 2001; Fletcher et al. 2015; Bonell et al. 2015) and, when time was available to set up projects (Bonell et al. 2015), conduct more meetings (Bell 2014) and embed systemic changes (Bond et al. 2001). Developing broadly based RAGs was challenging though (Bond et al. 2001). Recruiting and retaining external stakeholders like parents and governors were difficult (Mino 2003; Bell 2014; Bonell et al. 2010a), resulting in stakeholder absences or recruiting already engaged parents (Mino 2003; Bonell et al. 2010a). Less engaged students were not always involved due to time limitations (Bonell et al. 2010a).

Researchers concluded successful capacity-building to support *decision-making* about systemic change was attributed to the combination of RAG formation, external facilitators and school-specific data (Bond et al. 2001; Bonell et al. 2015). They thought facilitators’ presence supported stakeholders to form actions with clear intervention logic, considering iatrogenic effects too (Fletcher et al. 2015; Bonell et al. 2015), but they needed either school-based or youth work experience to do this effectively. Researchers agreed needs assessments were key to developing socially valid activities; however, other data sources such as audits (Bond et al. 2001; Bonell et al. 2015; Fletcher et al. 2015; Bonell et al. 2010a, b) and PhotoVoice (Davison et al. 2011) were only briefly discussed.

## Discussion

### Summary of Findings

The present review provides the first systematic attempt to synthesise evidence on types, processes and experiences of school-based health interventions developed through co-production with students aged 11–16. In answer to our first research question, three types of co-production were identified and modelled, differentiated by how capacity-building to support intervention development is conducted, and with whom. These were as follows: (1) external, which focused on generating capacity outside of the school by increasing facilitators’ knowledge of co-production processes; (2) individual-level, which involved upskilling students as researchers or group leaders and (3) system-level capacity-building, which entailed developing structural capacity through RAGs with multiple stakeholders.

Whilst this review has shown variability in co-production types and activities, there are shared functions. The review’s logic models (available online) show the functions found in co-produced interventions which are defined as follows. *Capacity-building* as how stakeholders are enabled to undertake intervention co-production within the school system. *Structure* and *recruitment* as how stakeholders are connected within the system, regardless of whether this includes the development of new or the use of existing activity settings or events. *Group development* as whether and how formalised processes are used to develop the social relationships between co-production stakeholders. How stakeholders achieve an understanding of health problem manifestation within schools, and decide on the target problems and solutions, are *problem-setting* and *problem-solving*, respectively. *Adoption* is whether and how further processes are used to accept proposed health activities, so *implementation* can focus on the delivery of health activities and how they saturate the context. *Evaluation* focuses on changes in outcomes.

To answer our second research question, the review also considered stakeholders’ experiences, particularly in terms of acceptability, feasibility and health activity decision-making. Predominantly, activities were deemed acceptable for all co-production types and for all those involved. There were variations in feasibility, for example, the conditions to support embedding a health-focused curriculum within educational settings, and difficulties in recruiting and retaining external stakeholders such as parents. These are not specific to co-production, but reflect wider issues identified in the school-based interventions literature (Langford et al. 2016). Nevertheless, they should be considered prior to future studies.

Issues with decision-making were found in all co-production types and expressed by all stakeholders apart from students. Constraining forces were both subtle, as facilitators manoeuvred students to more ‘acceptable’ ideas, and transparent, when school decision-makers refused student ideas. Future studies should assess this structural limiting of student’s agency and verify co-production has led to what is perceived as the most effective change activities possible within the context, before implementation. This could be achieved through extending process evaluation functions for co-produced interventions to include assessing the ‘social validity’ of decision-making and the emergent intervention plans with stakeholders, as in Bell (2014; et al. 2017). This would also temper the over-reliance on acceptability, as it is believed students rate projects highly because any involvement is better than the status quo, and they struggle to critically evaluate projects (Ozer et al. 2013).

### Conceptualising Co-production

Utilising core functions, rather than a single definition of co-production, allowed the research team to incorporate the complexity of the approach whilst avoiding ‘cobiquity’ (Williams et al. 2020). Functions supported complexity by allowing stakeholders and processes to vary between co-production examples, with the capacity-building function allowing a distinction between different types of co-production, whilst the key decision-making functions of problem-setting and solving (Bond et al. 2001; Hawe et al. 2000) avoided cobiquity through supporting the differentiation of co-production from other forms of collaboration. For example, ‘consultations’ aimed at asking stakeholder opinions on pre-set intervention ideas, and ‘participation as a means’ which invites involvement in pre-set activities such as delivering peer education (Baum 2015).

Defining co-production of interventions in terms of functions is in accordance with a growing perspective within complex systems thinking (Hawe et al. 2004, 2009). There has been a move from conceiving interventions in terms of standardised form with set activities, and hence conceptualising fidelity in terms of adherence to core activities. Rather, interventions are defined in terms of the functions that they seek to enact, and fidelity is understood more as the extent to which the intended theory of change is activated (Perez Jolles et al. 2019; Kemp 2016). This provides a vital progression to the field, where in recent years there has been an increase in the drive for both co-production in developing interventions (Moore and Evans 2017; Moore et al. 2019; Hawe et al. 2009) and the articulation of varied approaches to involving stakeholders (O’Cathain et al. 2019). There is wider learning about assessing fidelity at a functional rather than form level (Perez Jolles et al. 2019), with an absence of problem-setting and solving refuting the legitimacy of co-production.

### Implications for Prevention Science

Describing interventions in terms of their function rather than form has several other implications for prevention science. Standards of evidence in prevention science (Gottfredson et al. 2015) may need to broaden their conceptualisation of interventions to include co-produced interventions. Currently, interventions are described in terms of their content and articulating “core” components for replication purposes; however, incorporating descriptions of standardised key functions is needed. For example, this review found recruitment of diverse groups of stakeholders as a key co-production function, but this can be achieved through varied recruitment activities i.e. student nominations, applications and elections, staff nominations, and recruitment of existing school councils or classes. It is also recommended that future studies articulate interventions qualitatively and/or graphically through logic models to include co-production functions and their potential activities, as done here. In order to support this, there needs to be a debate about whether the functions and the interaction of processes with context can be graphically depicted in a better form. Attention to advancements in logic models may support this (Rehfuess et al. 2018; Rohwer et al. 2017; Mills et al. 2019).

Further, guidance (Gottfredson et al. 2015) states an account of intervention action and conceptual theories to express how mediators are activated and related to outcomes should be provided. This review demonstrated an absence of clear articulations of stakeholders’ theories of change for their decided health activities (apart from Bell 2014). This is problematic because it is important to ensure stakeholders’ assumptions have been correctly understood and delivered as intended. We propose the output of co-production should be a clear logic model of health activities. This can be utilised with stakeholders to assess the social validity of plans before implementation, and allow researchers to plan implementation assessments.

There remain vital questions as to whether co-production is effective. There is tentative evidence that the monocomponent, individual-level co-production type can lead to individual differences in socio-political skills, motivation to influence settings and participatory behaviour for co-production stakeholders (Ozer and Douglas 2013), but long-term changes in population health behaviours are unclear. Similarly, multicomponent interventions that utilise RAGs as well as standardised components have shown small effects on bullying but not aggression in school populations (Bonell et al. 2018), with our understanding of whether RAGs should be utilised unaccompanied, unclear. There are particular challenges with evaluating health outcomes for this contextually sensitive approach where the intervention may look distinct in different settings. Yet describing interventions in accordance with their function rather than form (Hawe et al. 2004, 2009; Perez Jolles et al. 2019; Kemp 2016) allows process and outcome evaluations to focus on the extent to which interventions activate the hypothesised theory of change, regardless of the activities conducted.

### Limitations of Included Studies

The primary limitation of studies was the lack of clarity on reporting. Nineteen studies were omitted as they lacked clear details on whether the intervention processes were within the remit for co-production. Even studies that met the inclusion criteria were variable and often opaque in their description of co-production. This may be partly explained by the complexity of co-production but may also be a result of an absence of comprehensive, standardised reporting guidance. A potential area for development, the functions here could be used to complement the reporting of co-produced interventions in the already existing GRIPP2 guidance for the reporting of patient and public involvement (Staniszewska et al. 2017), or the TIDieR template for intervention description and replication (Hoffmann et al. 2014).

Quality appraisal indicated the methodological strength of studies and identified limitations that might be redressed in future research (available online). For the trustworthiness of process evaluations, these included a lack of rigour in sampling and outlining analytical approaches, and the absence of grounding findings in process data. An issue with the usefulness of some process evaluations for this review was the paradox of not always involving students’ views in evaluating processes or privileging other adult stakeholders. This suggests future evaluations should draw on process evaluation guidance (Moore et al. 2014).

## Conclusion

This review is the first comprehensive synthesis of the types, processes and stakeholders’ experiences of co-produced school-based health interventions with students aged 11–16. Articulating and differentiating co-production types provides a useful step in understanding the nuances between them. Articulating processes highlight the core functions necessary to activate the underpinning theory of change and support the reporting of co-production. It can further assist in the conduct of process evaluations, as it has demonstrated key areas of acceptability, feasibility, decision-making and social validity. In addition to building our understanding of how co-produced interventions work in context, future research should conduct outcome evaluations to refine the evidence base for theoretical types and assess whether they can lead to demonstratable improvements in adolescent health outcomes.

## Supplementary Information

ESM 1 (DOCX 100 kb)

ESM 2 (DOC 65 kb)
