# Supplementary material for: Co-production as an Emerging Methodology for Developing School-Based Health Interventions with Students Aged 11–16: Systematic Review of Intervention Types, Theories and Processes and Thematic Synthesis of Stakeholders’ Experiences
Source: Prev Sci. 2020 Nov 25;22(4):475–91. doi: 10.1007/s11121-020-01182-8 (PMC8060205; doi:10.1007/s11121-020-01182-8)
Supplement: Supplementary file 1 — (DOCX 100 kb) [file 11121_2020_1182_MOESM1_ESM.docx]

# Supplementary Materials

**Table 2** Generic search strategy (adapted to the functionality of each database: Medline and PsycINFO (Ovid); Embase; ASSIA; and ERIC).

| Population/  Sample | (student* or pupil* or young* or youth* or teen* or adolescen* or child*).tw.  or students/ or exp high school students/ or exp junior high school students/ |
| --- | --- |
| Setting | AND (school*).tw. or exp Middle schools/ or exp high schools/ or exp junior high schools/ NOT schools, dental/ or schools, medical/ or schools, nursing/ or schools, pharmacy/ or schools, veterinary/ |
| Intervention | AND Intervention or program* or evaluat* or trial* or coproduc* or involv* or participat* or collabor* or decision-making* or decision making* or action* or engag* or council* or committee* or advis* or empower* |
| Research Type | AND (qualitative* or implement* or process or feasib* or pilot or case stud* or focus group* or focus-group* or interview* or grounded theor* or grounded-theor* or thematic analys* or framework analys* or discourse analy* or content analys* or hermeneutic* or phenomeno* or ethnograph* or interpretiv* or interpretativ* or realis*).tw. or exp qualitative research/ |
| Outcomes – | AND (tobacco OR smok* OR alcohol OR drink* OR drug* OR substance OR mental health OR wellbeing OR well-being OR depressi* OR anxi* OR emotion* OR life satisfaction OR violence OR bully* OR aggress*).tw. or exp tobacco smoking/ or exp drug usage/ or exp mental health/ or exp school violence/ |

Limiters - Published in English after 1986.

**Table 3** Quality Table (Assessment tool modified from the EPPI-Centre health promotion reviews)

| Capacity Building Type | Author & Year | 1. Rigour in Sampling | 2. Rigour in Data collection | 3. Rigour in Analysis | 4. Findings grounded in data. | 5. Breadth and Depth | 6. Privilege student perspective | 7.Trustworthiness (1-4) | 8.Relevance (5-6) |
| --- | --- | --- | --- | --- | --- | --- | --- | --- | --- |
| External | deLara 2000 | No SE data | No SE data | No SE data | No SE data | No SE data | No SE data | Low | Low |
| External | Paul et al 2010 | Yes, a few steps were taken | Yes, minimal steps were taken | No, not at all | Limited grounding and support | Limited breadth or depth | No | Low | Low |
| External | Tew 2010 | No SE data | No SE data | No SE data | No SE data | No SE data | No SE data | Low | Low |
| External | Paul et al 2012 | Can't tell | No, not at all | No, not at all | Limited grounding and support | Limited breadth or depth | Not at all | Low | Low |
| External | Vaughn et al 2013 | No SE data | No SE data | No SE data | No SE data | No SE data | No SE data | Low | Low |
| External | Voight 2015 | Yes, several steps were taken | Yes, a fairly thorough attempt was made | Yes, a fairly thorough attempt was made | Limited grounding and support | Good breadth but little depth | Somewhat | Medium | Medium |
| Individual-level | Jensen et al 2005 | Can't tell | Can't tell | Can't tell | Fair grounding and support | Good breadth but fair depth | Somewhat | Low | High |
| Individual-level | Simovska 2007 | Yes, several steps were taken | Yes, several steps were taken | Yes, several steps were taken | Good grounding and support | Good breadth but fair depth | Somewhat | High | Medium |
| Individual-level | Simovska and Jensen 2008 | Yes, several steps were taken | Yes, several steps were taken | Yes, several steps were taken | Good grounding and support | Good breadth but fair depth | No | High | Medium |
| Individual-level | Epstein 2007 | Yes, several steps were taken | Yes, a several steps were taken | Yes, several steps were taken | Good grounding and support | Fair Depth but very little breadth | A little | Medium | Medium |
| Individual-level | Soleimanpour et al 2008 | Can't Tell | Can't Tell | Can't Tell | Can't Tel | Limited breadth and depth | Can't tell | Low | Low |
| Individual-level | Youth In Focus 2002 | No SE data | No SE data | No SE data | No SE data | No SE data | No SE data | Low | Low |
| Individual-level | Ozer et al 2008 | Can't tell | Yes, a few steps were taken | Can't Tell | Good grounding and support | Good/fair breadth and depth | A little | Medium | Medium |
| Individual-level | Ozer et al 2010 | Can't Tell | Yes, a fairly thorough attempt was made | Can't Tell | Fair Grounding and Support | Good/fair breadth and depth | A little | Medium | Medium |
| Individual-level | Ozer et al 2013 | Yes, a fairly thorough attempt was made | Yes, a fairly thorough attempt was made | Yes, several steps were taken | Good Grounding and Support | Good/fair breadth and depth | Somewhat | High | High |
| Individual-level | Miller 2010 | Yes, several steps were taken | Yes, several steps were taken | Can’t tell | Limited grounding and support | Limited breadth or depth | Somewhat | Medium | Low |
| Individual-level | Goodnough 2014 | Yes, several steps were taken | Yes, a fairly thorough attempt was made | Yes, several steps were taken | Fair grounding and support | Fair depth but very little breadth | Somewhat | Medium | Medium |
| Individual-level | Shriberg et al 2017 | Yes, several steps were taken | Yes, minimal steps were taken | Yes, several steps were taken | Fair grounding and support | Good/fair breadth but very little depth | A lot | Medium | Medium |
| System-level | Bond et al 2001 and | Yes, a few steps were taken | Yes, a fairly thorough attempt was made | Not sure | Good grounding and support | Good breadth and depth | Not at all | Medium | Medium |
| System-level | Glover et al 2002 | No SE data | No SE data | No SE data | No SE data | No SE data | No SE data | Low | Low |
| System-level | Mino 2003 | Yes, several steps were taken | Yes, a fairly thorough attempt was made | No | Limited grounding | Good breadth but little depth | Don't know | Medium | Medium |
| System-level | Poulin and Nicholson 2005 | No SE data | No SE data | No SE data | No SE data | No SE data | No SE data | Low | Low |
| System-level | Bonell et al 2010a | Yes, a fairly thorough attempt was made | Yes, a fairly thorough attempt was made | Yes, a fairly thorough attempt was made | Good grounding and support | Good breadth and depth | Somewhat | High | High - |
| System-level | Bonell et al 2010b | Yes, a fairly thorough attempt was made | Yes, a fairly thorough attempt was made | Yes, a fairly thorough attempt was made | Good grounding and support | Good breadth and depth | Somewhat | High | High |
| System-level | Davison et al 2011 | Not sure | Not sure | No | Not sure | Limited breadth and depth | No | Low | Low |
| System-level | Hawe et al 2015 | No SE data | No SE data | No SE data | No SE data | No SE data | No SE data | Low | Low |
| System-level | Bell 2014 | Yes, several steps were taken | Yes, a fairly thorough attempt was made | Several steps were taken | Good grounding and support | Good breadth and depth | A little | Medium | Medium |
| System-level | Bell et al 2017 | Yes, several steps were taken | Yes, a fairly thorough attempt was made | Several steps were taken | Good grounding and support | Good breadth and depth | A little | Medium | Medium |
| System-level | Bonell et al 2015 | Yes, a fairly thorough attempt was made | Yes, a fairly thorough attempt was made | Yes, a fairly thorough attempt was made | Good grounding and support | Good breadth and depth | Somewhat | High | High |
| System-level | Fletcher et al 2015 | Yes, a fairly thorough attempt was made | Yes, a fairly thorough attempt was made | Yes, a fairly thorough attempt was made | Good grounding and support | Good breadth and depth | Somewhat | High | High |

SE – Stakeholders Experience

**Figure 2** Logic Model of Pathways for External Capacity-Building ( ) indicates number of studies

**Processes**

**Outcomes**

**Inputs**

**1. Capacity-Building**

*What:*

Knowledge of co-production theory and/or structuring framework

*Who:*

Researcher Facilitators (5)

Charity Worker Facilitator (1)

**8. Evaluation**

*Co-production Individuals:*

Pre and post socioemotional competencies (1)

*School-level Data:*

Pre and post Perception of school climate and pro-and antisocial behaviour (1)

Student behaviour incidents (1)

**7. Adoption and Implementation**

Adoption: Not detailed (3)

*Student ideas delivered to decision-makers:* School report (1); Student presentation (1); Staff use student picture (1);

Implementation: Assessed (0)

**2. Structure**

What: Core student groups (3);

Multiple stakeholders in problem-setting and solving processes inclusive of students (3), staff (3) and parents (1).

**3. Recruitment**

What: Staff nominations (4).

**6. Problem-Solving**

*How:* Stakeholder consensus discussions – Unstructured (1) or structured through: Socratic questioning (1); School opinion poll (1); or School problem pictures (1)

Researcher-led *-* brainstorming exercise plus analysis (1); researcher analysis of problem-setting data plus member checking (1)

**4. Group Development Tasks**

How: Ice breakers, agreeing ground rules, and discussing participation and confidentiality (1)

**5. Problem-Setting**

*How:* Student-led data collection to support discussions (1); Student group discussions (2).

Researcher-led school surveys (3), plus interviews and focus groups (1).

**Figure 3** Logic Model of Pathways for Individual-Level Capacity-Building for Co-production ( ) indicates number of studies

**Outcomes**

**Inputs**

**Processes**

**2. Structure**

*What:* Class Level (6); Small Group (3)

**1. Capacity-Building**

*What:* Development of co-production curricula/ training delivered to school stakeholders

*Curricula/Training Foci:*

Research Skills (6); Organisational Change (5); Student Leadership (2); Project Understanding (1)

*Who: Curricula/Training Delivery and Project Facilitation*

Youth workers delivering directly to students and facilitating on-site (4); youth workers delivering to both teachers and students for all adults to co-facilitate on-site (1); researchers delivering and facilitating with students (2); researchers training teachers who facilitate on-site (1); researcher delivering training to teachers and students for all adults to co-facilitate on-site (1)

**8. Evaluation**

*Co-production Individuals and controls:* Socio-political skills, motivation to influence school and community, participatory behaviour and perceived control (1)

**7. Adoption and Implementation**

Adoption: Not detailed (3);

*Student ideas delivered to decision-makers:* Presentations (6); seamless adoption (2); school decide adoption process (1).

Implementation: Assessed (0)

**3. Recruitment**

*How:* Teachers elect class (2); Students elect class (4); Staff nominations (1); Student applications (1); School Council (1)

**6. Problem-Solving**

*How:* Group Discussions (9) structured through: Problem-setting research (2); Facilitator questioning (1); Facilitator scaffolding (1)

**4. Group Development Tasks**

Ice breakers (3); communication skills (3); developing ground rules (1); active listening (1); goal setting (1).

**5. Problem-Setting**

*Topic Selection:* Students decide (6); Research team limit (2); Research team decide (1)

*How:* Students’ decide data collection method (7); Group discussion (1); Photography (1).

*Participant Data from:* Only recruited students (2); School peers (6) Staff (2); Students from other schools (1)

**Figure 4** Logic Model of Pathways for System-Level Capacity-Building for Co-production ( ) indicates number of studies

**Outcomes**

**1. Capacity-Building**

*What:* Development of a School Research Action Groups (RAG)

Project manuals (3);

Researcher collected data (6).

*Who:* Multiple school and external members.

Facilitators*:* Researcher (3); Employed expert (3); School staff (1)

**Processes**

**Inputs**

**8. Evaluation**

*Co-production Individuals:* participation and acceptability (1); acceptability and social validity (1)

*School-level Data:* mental health, smoking, and alcohol (1); bullying, substance use and mental, physical and sexual health, school engagement and social attachment (1); bullying victimisation and perpetration of violence and aggression, quality of life; psychological functioning and wellbeing, risk behaviours, NHS care, police contact and truancy(1); substance use and driving behaviour (1).

**7. Adoption and Implementation**

Adoption: RAG and facilitator remained (6); commitment from principal (1); funding allocated (4)

Implementation: Assessed (0)

**2. Structure**

RAGs (7)

**3. Recruitment**

*How:* School decided (3);

Election or self-nomination (2) ; Principal nomination (1); Staff and student nomination (1)

**6. Problem-Solving**

*How:* Group Discussion (7); theory, practice and research (2)

**5. Problem-Setting (understanding the school context and-prioritising problems)**

*Understanding School Context:* Needs assessment (6); Audits (4); Mapping hotspots (1); PhotoVoice and social network analysis (1); Group Discussion (1)

*Participants:* Students (6); Students and staff (1).

*Prioritising problems:* Group discussion (4) Ranking exercise (3), or through needs assessment (2).

Supplementary Data: school routine data (1); focus groups (1).

**4. Group Development Tasks**

*How:* Agreeing group rules and goals, and electing roles (2)

Group Development Tasks (2)
